# Dopaminergic Neurodegeneration in the Mouse Is Associated with Decrease of Viscoelasticity of Substantia Nigra Tissue

**DOI:** 10.1371/journal.pone.0161179

**Published:** 2016-08-15

**Authors:** Elisabeth G. Hain, Charlotte Klein, Tonia Munder, Juergen Braun, Kerstin Riek, Susanne Mueller, Ingolf Sack, Barbara Steiner

**Affiliations:** 1 Department of Neurology, Charité, University Medicine Berlin, Berlin, Germany; 2 Institute for Medical Informatics, Charité, University Medicine Berlin, Berlin, Germany; 3 Department of Radiology, Charité, University Medicine Berlin, Berlin, Germany; 4 Center for Stroke Research Berlin, Berlin, Germany; National Institutes of Health, UNITED STATES

## Abstract

The biomechanical properties of brain tissue are altered by histopathological changes due to neurodegenerative diseases like Parkinson's disease (PD). Such alterations can be measured by magnetic resonance elastography (MRE) as a non-invasive technique to determine viscoelastic parameters of the brain. Until now, the correlation between histopathological mechanisms and observed alterations in tissue viscoelasticity in neurodegenerative diseases is still not completely understood. Thus, the objective of this study was to evaluate (1) the validity of MRE to detect viscoelastic changes in small and specific brain regions: the substantia nigra (SN), midbrain and hippocampus in a mouse model of PD, and (2) if the induced dopaminergic neurodegeneration and inflammation in the SN is reflected by local changes in viscoelasticity. Therefore, MRE measurements of the SN, midbrain and hippocampus were performed in adult female mice before and at five time points after 1-methyl-4-phenyl-1,2,3,6-tetrahydropyridin hydrochloride (MPTP) treatment specifically lesioning dopaminergic neurons in the SN. At each time point, additional mice were utilized for histological analysis of the SN. After treatment cessation, we observed opposed viscoelastic changes in the midbrain, hippocampus and SN with the midbrain showing a gradual rise and the hippocampus a distinct transient increase of viscous and elastic parameters, while viscosity and–to a lesser extent—elasticity in the SN decreased over time. The decrease in viscosity and elasticity in the SN was paralleled by a reduced number of neurons due to the MPTP-induced neurodegeneration. In conclusion, MRE is highly sensitive to detect local viscoelastic changes in specific and even small brain regions. Moreover, we confirmed that neuronal cells likely constitute the backbone of the adult brain mainly accounting for its viscoelasticity. Therefore, MRE could be established as a new potential instrument for clinical evaluation and diagnostics of neurodegenerative diseases.

## Introduction

The macroscopic biomechanical properties of *in vivo* brain tissue are influenced by the cellular composition of the brain given by the number of neurons and glial cells as well as their interactions with the extracellular matrix [1–3]. This composition varies in diverse brain regions and under pathological conditions so that histological differences may be reflected in the biomechanical properties of tissue and can be represented in viscoelastic quantities. Therefore, alterations in viscoelasticity can be considered to be a potential instrument for clinical evaluation and diagnostics.

Magnetic resonance elastography (MRE) attracted attention as an appropriate medical imaging technique to assess biomechanical properties of brain tissue non-invasively and *in vivo* [4]. Biomechanical constants of soft tissues are measured by inducing shear waves and processing the MR images of the propagating shear waves to calculate quantitative values of viscoelasticity such as the complex shear modulus G* [4,5]. G* contains the storage modulus G' and the loss modulus G''. G' gives information about the elasticity of the tissue, which is determined by the number and type of cells in the network. In contrast, G'' gives information about the viscous, dampening properties of the tissue, which depend on the geometry of the network including bonds and branching.

In human MRE studies, it has been found that brain viscoelasticity is reduced during aging [6,7] and under pathological conditions like multiple sclerosis (MS) [8,9], normal pressure hydrocephalus [10], Alzheimer's disease (AD) [11], frontotemporal dementia [12], Glioblastoma [13] and progressive supranuclear palsy [14]. However, the histopathological mechanisms underlying the observed alterations in tissue viscoelasticity are still not completely understood.

With the use of animal models, first steps have been made to elucidate the link between MRE parameters and histology. Millward, Riek and colleagues revealed a correlation between the degree of inflammation, mediated by T-cells and macrophages/microglia, and the viscoelastic constants in a mouse model of MS [15,16]. Schregel and co-workers underlined the decrease of elasticity in a different MS mouse model caused by demyelination and changed extracellular matrix configuration [3].

Aside from inflammation, neuronal alterations have also been observed to play an important role in changed viscoelastic parameters in the MRE. After middle cerebral artery occlusion in mice, the depleted density of neurons correlated directly with reduced elastic properties in the affected brain hemisphere [17]. In addition, mouse models for AD and Parkinson’s disease (PD), have been investigated as well. A softening of brain tissue has been observed by MRE in APP-PS1 AD mice [18], but correlating histopathological analyses with particular regard to local changes correlating to region specific changes in MRE are missing. Changes in viscoelasticity and correlating histopathological mechanisms have been observed in previous animal studies in the whole brain [16], in one hemisphere [17] or the cerebellum [15], whereas such investigations in smaller brain regions are still not completed. In the 1-methyl-4-phenyl-1,2,3,6-tetrahydropyridine hydrochloride (MPTP) mouse model reproducing PD-like histopathology, a transient rise in viscoelasticity in the hippocampus has been observed. This was paralleled by a higher density of newly generated neurons, arising from a reactively generated precursor cell population [1]. However, the lesioned substantia nigra (SN) as the mainly affected structure in PD and its models has not been investigated yet in detail. In the work of Klein et al., basic principles in the relation between changes in the number of neurons under neurodegenerative conditions and MRE-measured viscoelastic properties using the MPTP mouse model for PD has been established [1].

PD, however, is initially and mainly characterized by the loss of dopaminergic neurons in the SN, a small region in the midbrain with synaptic connections to the surrounding basal ganglia and beyond [19]. The neurotoxin MPTP is an established animal model for the histopathology seen in PD patients [20] due to its ability to selectively lesion dopaminergic cells in the SN, which is also accompanied by inflammatory reactions [21,22]. Up to now, Lipp and co-workers demonstrated a reduction of elasticity in the lentiform nucleus as part of the midbrain in humans with PD, whereas the viscoelastic properties of the whole brain were unaffected [14]. However, region specific changes of viscoelasticity due to aging and pathological conditions like neurodegenerative diseases and the analysis of underlying histopathological alterations is still lacking. Thus, we applied the MRE setup to MPTP-lesioned mice to investigate, if acquired viscoelastic parameters are altered in affected areas: the SN, midbrain and hippocampus. Furthermore, we investigated, if changes in MRE parameters in the SN correlate with neurodegenerative and inflammatory processes and if we can confirm that MRE is feasible to selectively detect local pathological alterations in neurodegenerative diseases. This would add to the establishment of MRE as clinical evaluation tool.

## Materials and Methods

### Animal Treatment

All animal experiments were approved by the local animal ethics committee (Landesamt für Gesundheit und Soziales, Berlin, Germany) and carried out in accordance with the European Communities Council directive of 22 September 2010 (10/63/EU). In total, 35 female eight to ten weeks old C57Bl/6N mice were group-housed in a temperature- and humidity-controlled colony room with a light/dark cycle of 12/12 h and unrestricted access to food and water.

**Fig 1:**
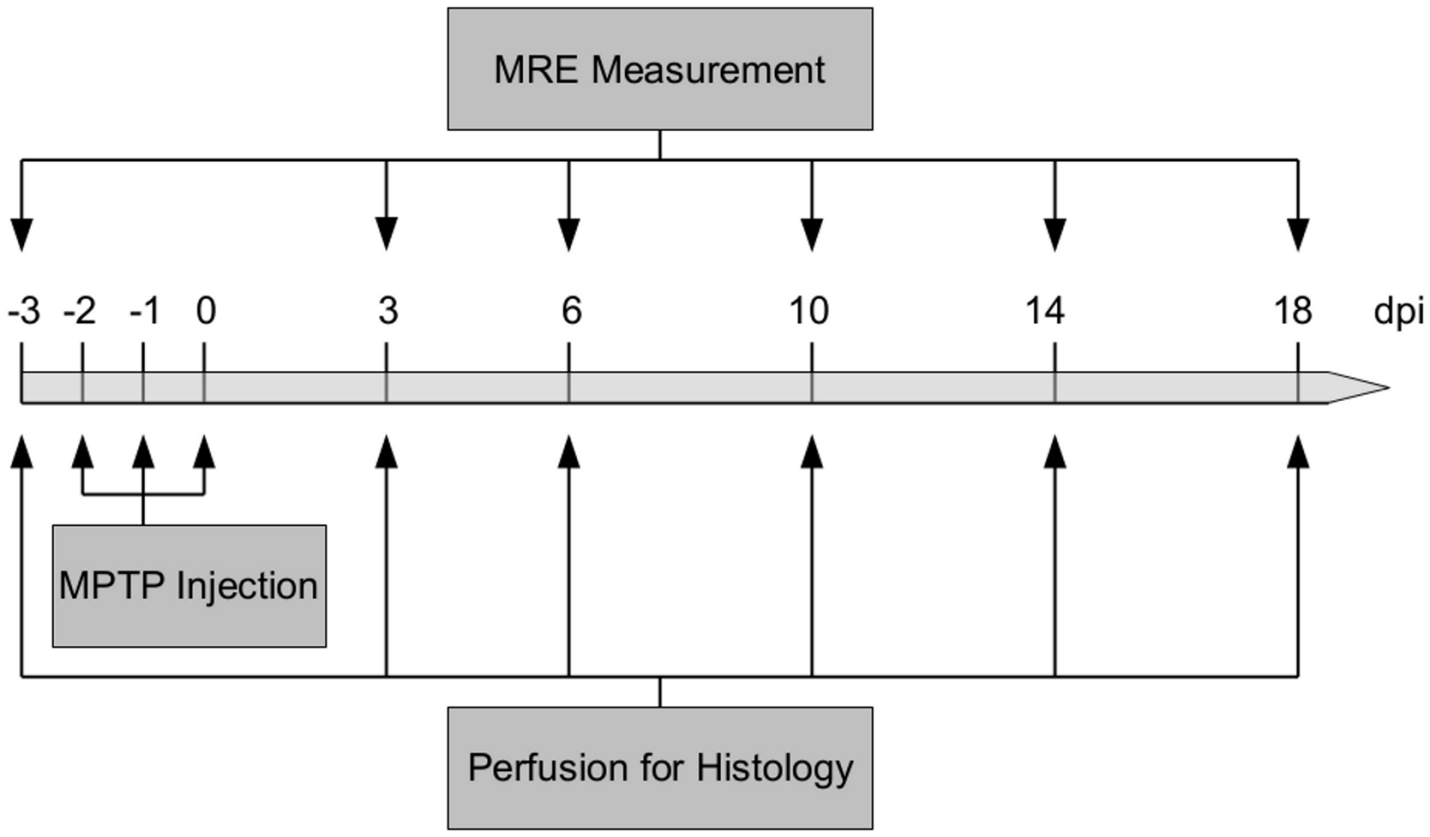
Timeline of the experimental procedure. The timeline of MPTP injections, time points of MRE measurement and brain perfusions for histological analyses.

Animals were randomly divided into seven groups of n = 5. All animals, except the histological counterparts for the baseline measurement, were treated intraperitoneally with MPTP (Sigma Aldrich, Steinheim, Germany), dissolved in 0.9% NaCl, with a concentration of 20 mg/kg bodyweight on three consecutive days. One group (n = 5) underwent MRE-imaging the day before MPTP treatment started (-3 days post-injection (dpi)) as baseline and three, six, ten, 14 and 18 days after the last MPTP injection (3, 6, 10, 14, 18dpi). At each time point (six in total), animals of the corresponding histological group (n = 5 for each time point) were perfused. A timeline of the experimental procedure is given in Fig 1 and Table 1.

**Table 1:** Experimental design.

Post-injection day	Performance
**-3**	**MRE measurement or perfusion for histology**
**-2**	**MPTP injection**
**-1**	**MPTP injection**
**0**	**MPTP injection**
**3**	**MRE measurement or perfusion for histology**
**6**	**MRE measurement or perfusion for histology**
**10**	**MRE measurement or perfusion for histology**
**14**	**MRE measurement or perfusion for histology**
**18**	**MRE measurement or perfusion for histology**

### Perfusion and Tissue Preparation

At each time point of MRE measurement, the corresponding histological group of animals were deeply anaesthetized with Ketamine/Xylacine (10% Ketamine hydrochloride, WDT; 2% Rompun, Provet AG) and transcardially perfused with phosphate buffered saline (PBS) and 4% paraformaldehyde (PFA). Brains were dissected carefully, postfixed in 4% PFA for 24 h and dehydrated with 30% sucrose solution for 48 h. Then they were frozen in 2-methylbutane (Sigma-Aldrich, Steinheim, Germany) cooled with liquid nitrogen, sliced in 40 μm thick coronal sections using a Leica CM 1850 UV cryostat and stored in cryoprotectant solution until histological stainings were performed.

### Immunohistochemistry

For immunohistochemistry, a well-established staining protocol was followed [23,24]. Briefly, a one-in-six free-floating brain section series of each mouse was pre-treated with 0.6% H_2_O_2_ and donkey serum-enriched PBS (PBS+) before being incubated with the first antibody anti-Tyrosinhydroxylase (TH; mouse 1:10000, Sigma-Aldrich) or anti-ionized calcium-binding adapter molecule 1 (Iba-1; rat 1:1000, Wako) at 4°C overnight. On the next day, brain sections were first pre-treated with PBS+ for background blocking and then incubated with the secondary biotinylated antibody (anti-mouse or anti-rat, 1:250, Dianova) at room temperature for two hours. Then, ABC solution (Vectastain Elite ABC Kit, Vector Laboratories) was applied, before the formed streptavidin-peroxidase complex was visualized by 3,3'-diaminobenzidine (DAB, Sigma-Aldrich)-nickel staining. Finally, stained sections were mounted on microscope slides and coverslipped.

To determine the total cell number in each region of interest (SN, midbrain and hippocampus), a separate one-in-twelve series of brain sections of two mice per group was stained with the fluorochrome 4’,6-diamidino-2-phenylindole (DAPI), which binds to the DNA thereby labeling cell nuclei. Sections were incubated with PBS-diluted DAPI (1:1000, Thermo Scientific) for 7 min and afterwards mounted on microscope slides and coverslipped.

### Cell Quantification

In total, four stained brain slices of each mouse were analyzed for TH+ cells in the SN, including pars compacta and pars reticulata. Cells were manually counted under the 40x objective of an Axioskop HB50/AC light microscope (Zeiss, Germany) and multiplied by six to obtain an estimated absolute number of cells per SN.

For quantification of DAPI-stained cell nuclei in all regions of interest and Iba-1+ cells in the SN, the Stereo Investigator (MBF Bioscience) and a Leica DMRE microscope were used. The region of interest was defined with the 5x objective. Actual counting was done with the 40x oil objective. For quantification of DAPI-stained cell nuclei, two sections in an interval of twelve were counted by using a sampling grid size of 250x200 μm in the SN and 600x600μm in the midbrain and hippocampus. Iba-1 positive cells were counted in four sections in an interval of six by using a sampling grid size of 150x120 μm. In all brain regions, a counting frame of 60x60 μm without guard dissector height was used. Cells were counted when cell bodies became sharp in their widest extent. The absolute number of cells per brain regionwas automatically calculated based on the counted cell number, slice interval, counting frame size, sampling grid size and slice thickness.

### Magnetic Resonance Elastography (MRE)

**Fig 2:**
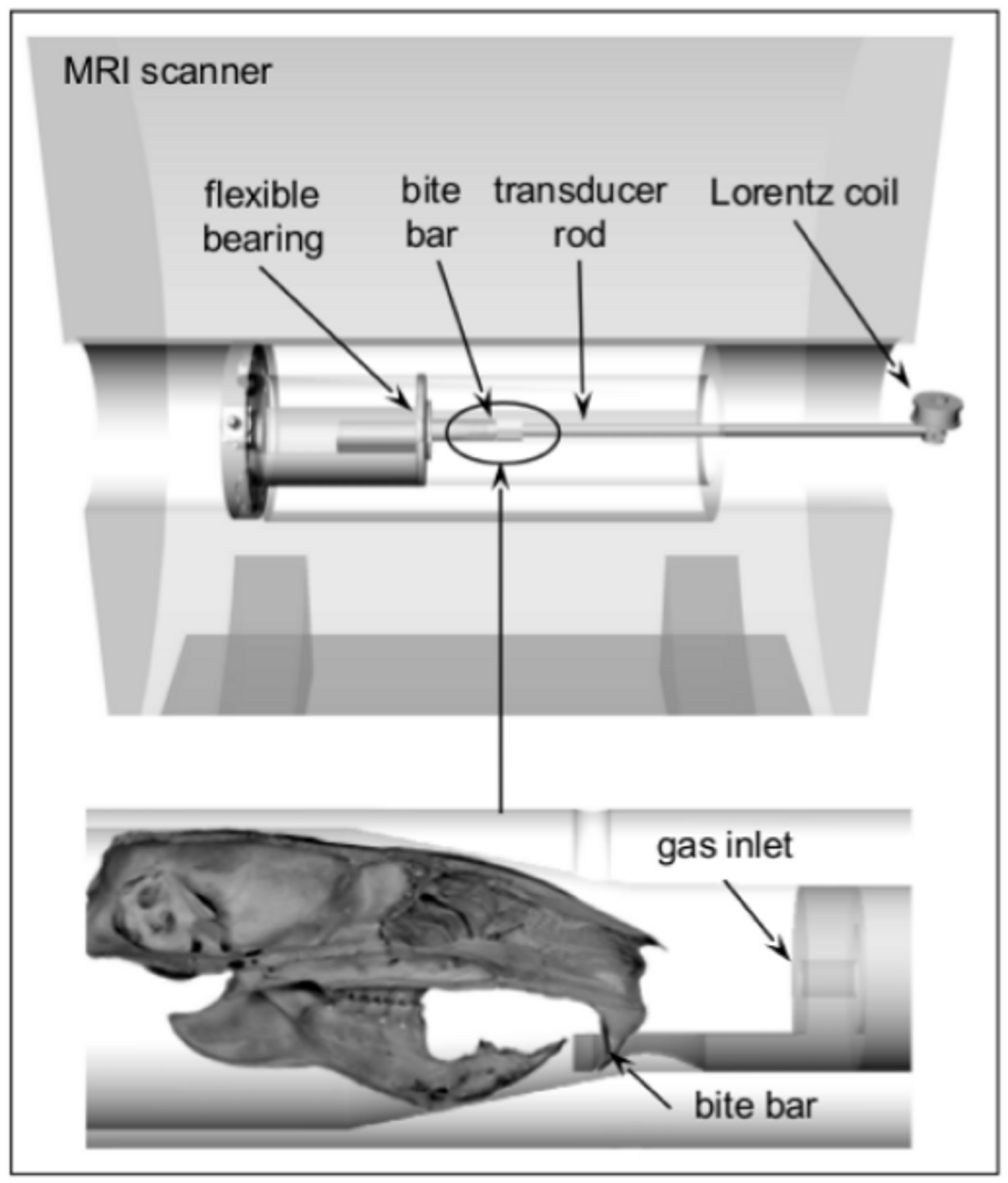
Schematic of the mouse MRE apparatus.

All measurements were realized on a 7-Tesla MR Imaging (MRI) scanner (Bruker PharmaScan 70/16, Ettlingen, Germany) with a 20 mm diameter 1H-RF-quadrature mouse head coil and using ParaVision 4.0 software. As illustrated in Fig 2, shear waves into the mouse brain were induced by using a moveable bite bar transducer, linked with a carbon fiber piston to an electromagnetic coil as the source of vibration. During the MRE session, mice were anaesthetized with isoflurane/oxygen. The transducer was gimballed through a rubber bearing and retaining bracket at the temperature-controlled mouse bed. This setup was held in the middle of the magnet bore of the MRI scanner by a plastic disc. Vibrations were induced by applying a sinusoidal electric current of 900 Hz frequency to an air-cooled Lorentz coil in the fringe area of the MRI scanner and were initialized by a trigger pulse from the control unit of the scanner, while the timing was defined by a customized FLASH sequence. Frequency, amplitude and number of sinusoidal oscillation cycles were controlled by an arbitrary function generator connected via an audio amplifier to the driving coil. The main polarization of the vibration was transverse to the principal axis of the magnet field, with amplitudes in the order of tens of micrometers.

The MRE data were acquired in one 2 mm transverse slice in which all regions of interests (ROI) could be analyzed. The imaging sequence was modified for MRE by sinusoidal motion sensitizing gradient (MSG) in the through-plane direction, as described elsewhere [16]. The MSG strength was 285 mT/m with a frequency of 900 Hz and nine periods. Phase difference images were calculated from two images differing in the sign of the MSG to compensate for static phase contributions. Additional imaging parameters were: a 128x128 matrix, 25 mm FoV, 14.3 ms echo time, 116.2 ms repetition time, eight dynamic scans over a vibration period and an acquisition time of 20 min.

**Fig 3:**
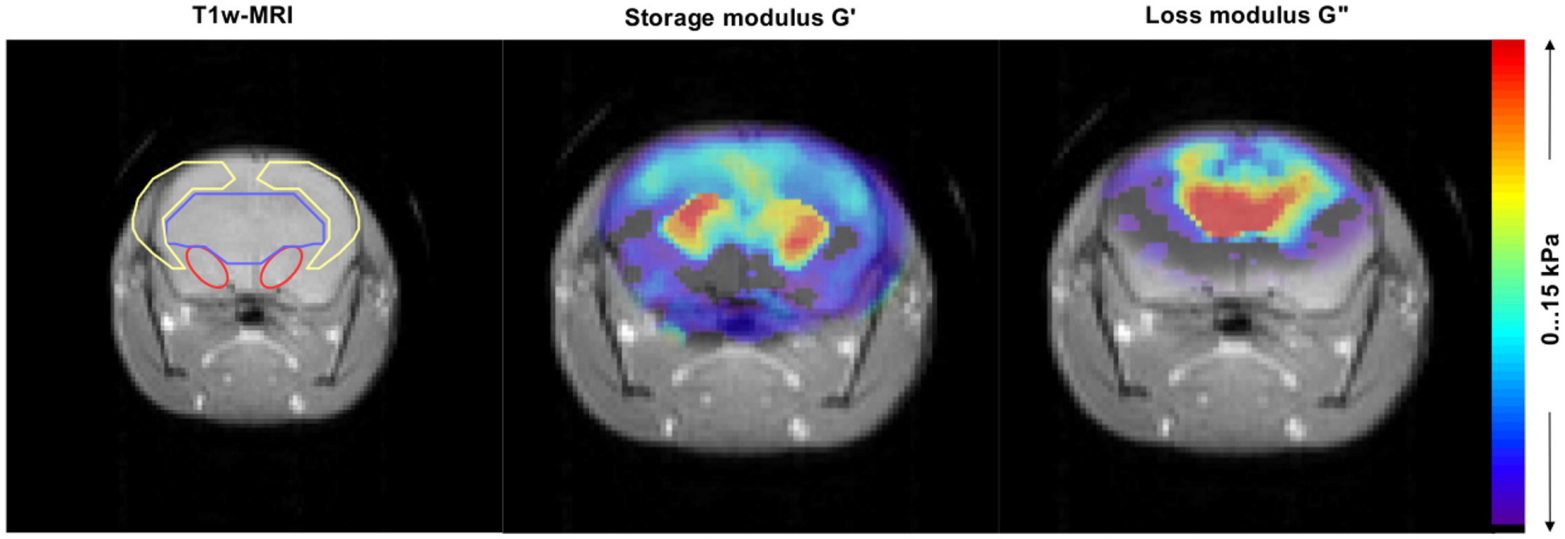
Representative images of MRI signal and complex modulus map of G' and G''. Regions of interest: substantia nigra (red line), midbrain (blue line) and hippocampus (yellow line) were marked in T1w-MRI.

Complex wave images according to the harmonic drive frequency were estimated by temporal Fourier transformation of the unfolded phase-difference images and filtered for suppressing noise and compression wave components [16,25]. An algebraic Helmholtz inversion was applied to the pre-processed 2D scalar wave fields calculating the complex shear modulus G* [26]. Then, G* was spatially averaged over the SN, midbrain and hippocampus of both hemispheres as ROIs, which were manually segmented by delineating its anatomical structure from T1w-MR images (Fig 3). Values of the averaged G* contain the real part of G*: storage modulus G', and the imaginary part of G*: loss modulus G'', representing the elasticity and viscosity of tissue, respectively.

### Statistical Analysis

All statistical analyses were performed by using SPSS Statistics19 for Windows and GraphPad Prism 5. The homogeneity of variance was tested by Levene test. One-way ANOVA was performed the data from the quantification of TH+ and Iba1+ cells and one-way repeated measures (RM) ANOVA for MRE data. The data from the DAPI counts were not statistically evaluated, because only n = 2 per time point were quantified. Pairwise comparisons were done using the Bonferroni test in case of a significant ANOVA. The level of statistical significance was set at p≤0.05. All data are shown as mean values with standard error of the mean (SEM). Graphs were generated using GraphPad Prism 5.

## Results

**Fig 4:**
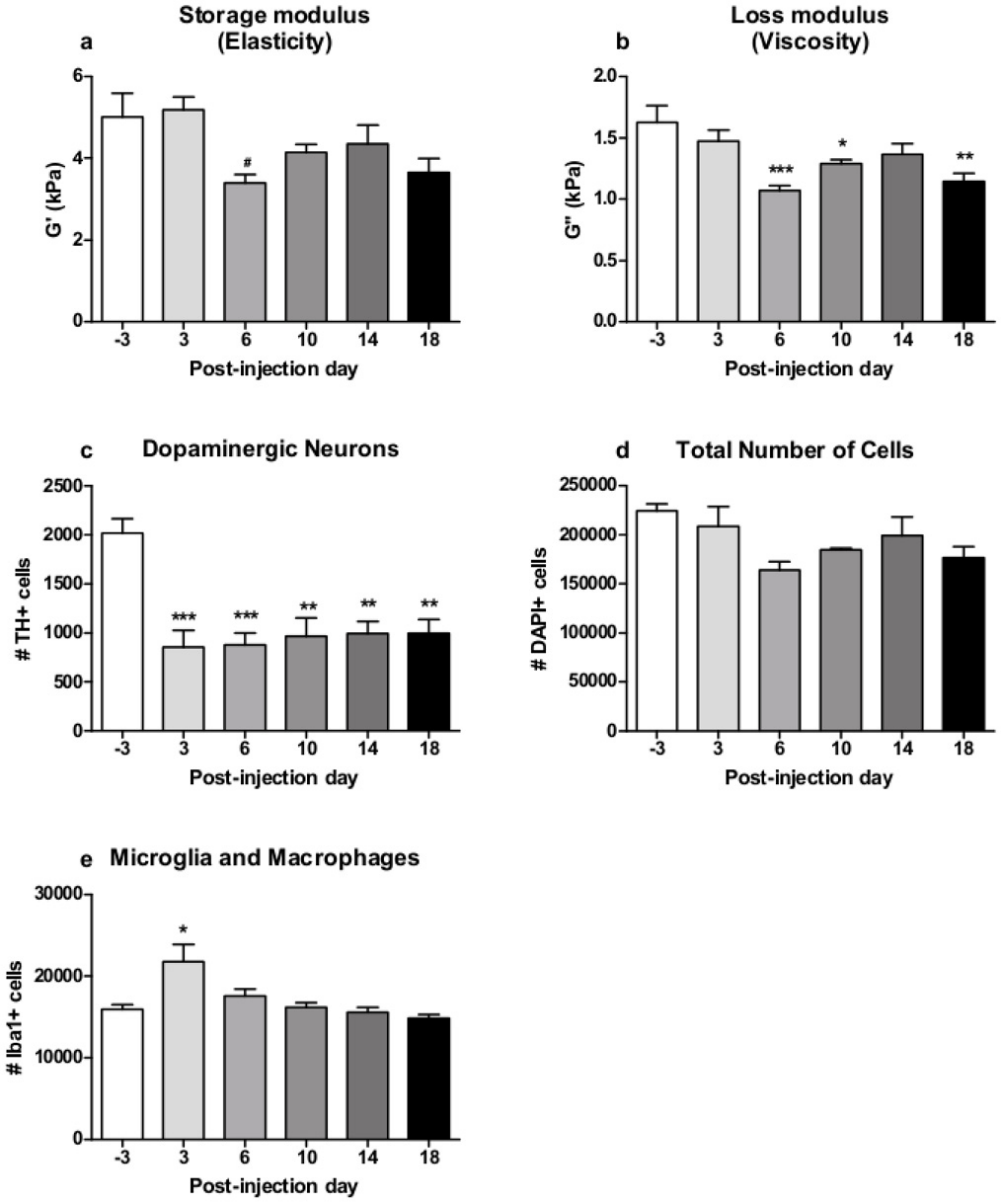
Results of MRE measurements and histological cell counts in the substantia nigra. MPTP induced a significant reduction in MRE elasticity (a) and viscosity (b) in the substantia nigra (mean±SEM, n(-3,3,6,10,14,18dpi) = 5). DAB-stained brain sections showed an immediate significant drop in TH+ dopaminergic neurons in the substantia nigra after MPTP treatment (c) (mean±SEM, n(-3dpi) = 4, n(3,6,10,14,18dpi) = 5). DAPI-stained cell amount was decreased by MPTP (d) (mean±SEM, n(-3,3,6,10,14,18dpi) = 2, no statistical analysis). Initially, the amount of Iba1+ microglia and macrophages was significantly raised after MPTP treatment, but ceased over time (e) (mean±SEM, n(-3dpi) = 4, n(3,6,10,14,18dpi) = 5). * vs. -3dpi, *p≤0.05, **p≤0.01, ***p≤0.001. ^#^ vs. 3dpi, ^#^ p≤0.05.

**Fig 5:**
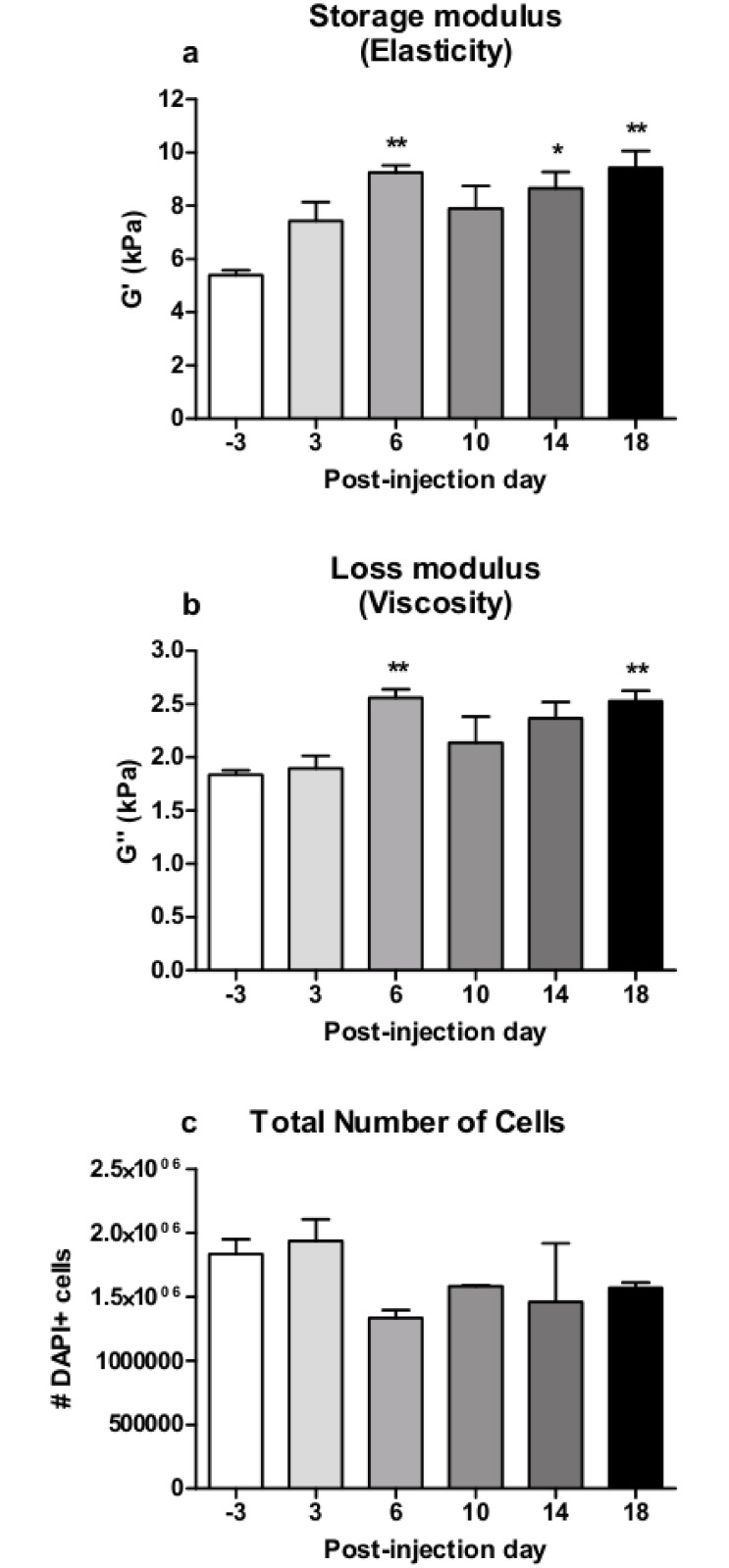
Results of MRE measurement and histological cell count in the midbrain. MPTP induced a significant increase of MRE elasticity (a) and viscosity (b) in the midbrain (mean±SEM, n(-3,3,6,10,14,18dpi = 5). DAPI-stained brain sections showed a reduction following MPTP-treatment (c) (mean±SEM, n(-3,3,6,10,14,18dpi) = 2, no statistical analysis). * vs. -3dpi, *p≤0.05, **p≤0.01.

**Fig 6:**
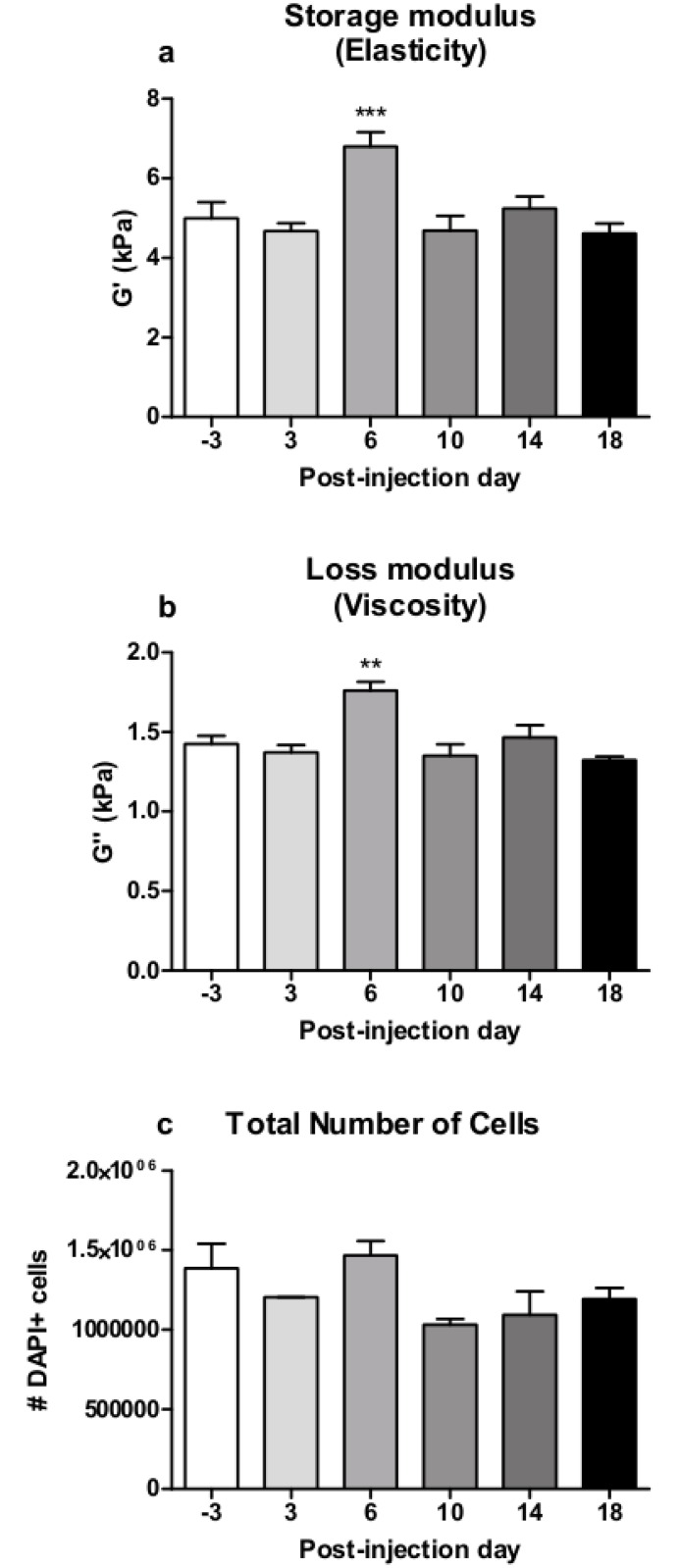
Results of MRE measurement and histological cell count in the hippocampus. MPTP induced a transient increase of elasticity (a) and viscosity (b) in the hippocampus at 6dpi (mean±SEM, n(-3,3,6,10,14,18dpi = 5). Quantification of DAPI-stained cells showed an elevated amount at 6dpi (mean±SEM, n(-3,3,6,10,14,18dpi) = 2, no statistical analysis). * vs. -3dpi, **p≤0.01, ***p≤0.001.

Initially, MRE measurement was performed the day before MPTP treatment started to generate baseline data of healthy brain tissue. Sessions were repeated three, six, ten, 14 and 18 days after the last MPTP injection and processed for the SN, midbrain and hippocampus. The MRE results are presented together with the results of the histological analysis of each brain region in Figs 4, 5 and 6.

One-way ANOVA of the MRE parameters at -3dpi revealed a significant difference in basic viscosity between the various brain areas (F(2,14) = 5.396, p≤0.05). Post-hoc pairwise comparison using the Bonferroni test showed a significant higher viscosity in the midbrain (1.836 ±0.043 kPa) than in the hippocampus (1.426 ±0.051 kPa) (p≤0.05).

### MPTP induces a decrease of viscosity and elasticity in the SN

In the SN, MRE generated baseline values with mean (±SEM) of 5.012 (±0.578) kPa and 1.627 (±0.137) kPa for G' and G'', respectively. A one-way RM ANOVA revealed a significant effect in the storage modulus G' (F(5,29) = 4.274, p≤0.01) and loss modulus G' (F(5,29) = 8.350, p≤0.001) following MPTP treatment, which reflects alterations in the elastic and viscous properties of the SN. Post-hoc pairwise comparison using the Bonferroni test showed a significant decrease in G’ (3dpi vs. 6dpi: p≤0.05) (Fig 4a) and G'' (-3dpi vs. 10dpi: p≤0.05, -3dpi vs. 18dpi: p≤0.01, -3dpi vs. 6dpi: p≤0.001) (Fig 4b), indicating a more reduced viscosity than elasticity in the SN following MPTP treatment with a slight restoration over time.

### MPTP induces an increase of viscosity and elasticity in the midbrain

Mean values (±SEM) of G' and G'' were 5.397 (±0.190) kPa and 1.836 (±0.043) kPa in the midbrain at -3dpi. The one-way RM ANOVA revealed a significant effect of MPTP treatment on storage modulus G' (F(5,29) = 6.702, p≤0.001) and loss modulus G' (F(5,29) = 6.895, p≤0.001) in the midbrain over time. A significant increase in G’ (-3dpi vs. 14dpi: p≤0.05, -3dpi vs. 6dpi and 18dpi: p≤0.01) (Fig 5a) and G'' (-3dpi vs. 6dpi and 18dpi: p≤0.01) (Fig 5b) was observed as the post-hoc pairwise comparison showed. This indicates tissue stiffening in the midbrain after MPTP treatment.

### MPTP induces a transient increase of viscosity and elasticity in the hippocampus

At baseline, mean values (±SEM) were 4.997 (±0.402) kPa and 1.426 (±0.52) kPa for G' and G'', respectively. The One-way RM ANOVA showed a significant effect of MPTP treatment on the storage modulus G' (F(5,29) = 11.75, p≤0.001) and loss modulus G' (F(5,29) = 8.075, p≤0.001) in the hippocampus over time. Here, post-hoc pairwise comparison revealed a significant increase in G’ (-3dpi vs. 6dpi: p≤0.001) (Fig 6a) and G'' (-3dpi vs.6dpi: p≤0.01) (Fig 6b), indicating a transient tissue stiffening in the hippocampus six days after treatment cessation.

### MPTP induces dopaminergic neurodegeneration in the SN

**Fig 7:**
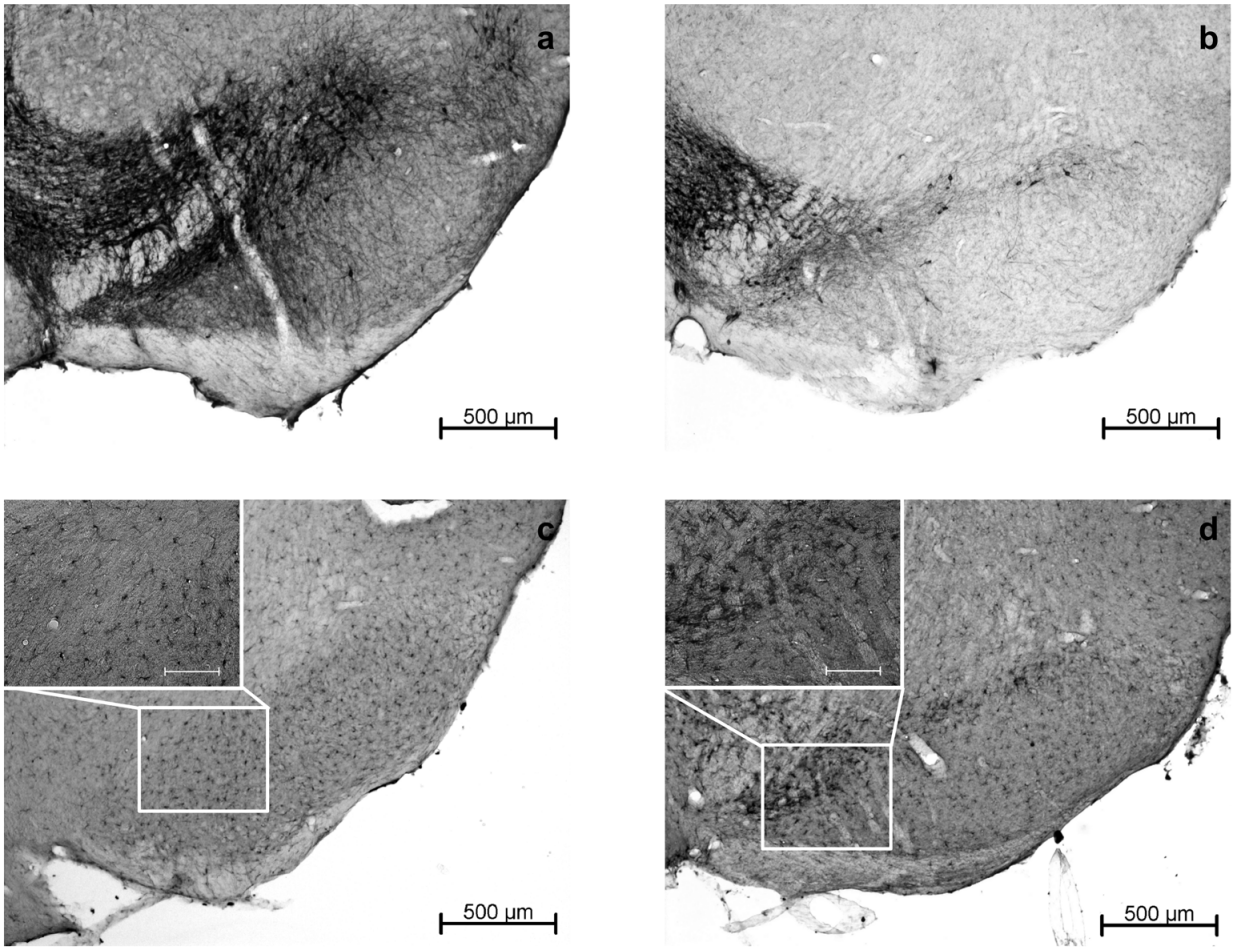
Representative images of DAB–stained brain slices showing the substantia nigra. TH+ cells at baseline at -3dpi (a) and directly after MPTP treatment at 3dpi (b) at 50x magnification, indicating a severe loss of dopaminergic neurons induced by MPTP. Iba-1+ at -3dpi (c) and 3dpi (d) at 50x magnification with detail in 200x magnification (scale bar 100 μm), showing a reactive increase in the number of microglia and macrophages in the substantia nigra immediately after MPTP treatment.

A one-way ANOVA revealed a strong effect of MPTP treatment on the number of TH+ in the SN (F(5,28) = 7.499, p≤0.001). Pairwise comparison showed that the number of TH+ dopaminergic neurons was decreased by MPTP at all time points in comparison to baseline level (-3dpi vs. 3dpi and 6dpi: p≤0.001, -3dpi vs. 10dpi, 14dpi and 18dpi: p≤0.01) (Fig 4c). The immediate reduction of TH+ dopaminergic neurons following MPTP treatment was approximately 57% with mean values declining from 2018 at -3dpi to 856 at 3dpi, respectively. The deficit of dopaminergic neurons persisted at least until the last time point at 18dpi investigated here. Representative images of TH+ cells before and after MPTP treatment are shown in Fig 7a and 7b.

### MPTP induces a reduction of total cell amount in the SN and midbrain and a transient rise in the hippocampus

In the SN, the quantification of DAPI+ cells (n = 2) revealed a decrease in the total amount of cells from 224700 (±6967) cells at -3dpi to 164083 (±8417) cells at 6dpi following MPTP-treatment. After that, a transient slight restoration over time can be observed (Fig 4d).

The DAPI+ cell count (n = 2) in the midbrain revealed a decrease in the total cell amount from 1834800 (±116400) to 1333800 (±60600) cells at -6dpi (Fig 5c).

In the hippocampus, the DAPI+ cell quantification (n = 2) revealed a total amount of 1385400 (±155400) cells at -3dpi and a transient rise at 6dpi up to 1468200 (±89400) cells (Fig 6c).

### MPTP induces a transient increase of microglia and macrophages in the SN

The analysis of Iba1+ cell counts revealed a significant effect of MPTP treatment on the number of microglia and macrophages in the SN as a sign for a local inflammatory reaction (F(5,28) = 5.706, p≤0.01). Pairwise comparison showed that the amount of Iba+ cells directly after MPTP treatment (3dpi) was significantly higher than at baseline (-3dpi vs. 3dpi: p≤0.05), whereas no significant difference in the amount of Iba-1+ cells was observed between baseline and later time points from 6dpi to the end of the study (Fig 4d). MPTP induced a transient inflammatory response, which is reflected here by increased numbers of microglia in the SN at 3dpi (21732 cells), that ceased over time until baseline levels (approximately 15000 cells) are reached again at 18dpi. Representative images of Iba+ cells before and after MPTP treatment are shown in Fig 7c and 7d.

## Discussion

In this study, we demonstrate how viscosity and elasticity in various brain areas change in response to MPTP treatment and that the significant reduction of dopaminergic neurons in the SN in a mouse model for a neurodegenerative disease is reflected in the decrease of viscosity and–to a lesser extent–elasticity. Hence, the MRE setup is viable to detect viscous and elastic alterations even in small brain areas.

The neurotoxin MPTP primarily affects dopaminergic neurons and is therefore an established animal model for the histopathology seen in PD patients [19,20]. Besides the SN, the MPTP mouse model is also used for investigations in the midbrain [27] and hippocampus [24] to elucidate the effects of nigral dopaminergic neurodegeneration in other specific brain regions related to the pathology of PD. According to the fact that PD (and its animal model) includes the neurodegenerative affection of different areas in the brain, e.g. reduced neural precursor cells caused by dopamine denervation in the subgranular zone of the hippocampus [28], such extra-nigral histopathological changes were correlated to region specific changes in viscoelasticity detected by MRE [1].

In the SN, we observed a significant decrease in viscosity and–to a lesser extent—elasticity six days after treatment cessation followed by a slight but non-significant restoration over time. MPTP seem to primarily affect the geometry of the cellular network in the SN and secondarily the cell density. This is comprehensible considering the structure of the SN, with dopaminergic cell bodies and many cellular processes, which are substantially reduced after MPTP treatment (see Fig 7a and 7b for comparison). Our findings are in line with an observed tissue softening in other animal and human studies of pathological conditions [8,15,17].

Surprisingly, in contrast to the SN, calculated MRE parameters of the midbrain were significantly higher after treatment compared to healthy baseline values. This rather indicates an increase in tissue stiffness in response to MPTP. The decreased total amount of DAPI+ cells does not correlate with this finding. Up to now, it is known that MPTP treatment does not lead to dopaminergic neurodegeneration in the midbrain in contrast to the SN [27]. The biomechanical properties of tissue are not only determined by individual cell types and cellular density but also by the complexity of the cellular network, which depends on the degree of cross-linking and branching, and the interaction with the extracellular matrix [1–3]. The discrepancy between midbrain MRE and histology data may therefore be attributed to other processes following MPTP treatment than the ones examined here, as the midbrain is a larger and more complex area with several core regions and white matter tracts than the SN and hippocampus and cannot be narrowed down to only neuronal cells. Though MRE sensitively detects local tissue alterations, the specificity of the method is not yet established in detail.

Interestingly, a higher basic viscosity was observed in the midbrain compared to the hippocampus in healthy yet untreated mice at -3dpi. As mentioned above, the midbrain tissue exhibits more complex network geometry with diverse core regions and white matter tracts. In humans, white matter has been shown to be stiffer than gray matter [29,30], which is in line with our observed higher viscosity in the midbrain.

In the hippocampus we observed a transient rise of the storage and loss modulus as described in our previous study [1]. In correlation to that, the total amount of cells was also transiently elevated at 6dpi. This underlines the histological findings of Klein and colleagues, showing increased stiffness in the hippocampus following MPTP treatment. This was correlated to a higher percentage of newly generated neurons resulting from a reactively enhanced precursor cell proliferation [1].

The significant reduction of G' and G'' in the SN at 6dpi without full restoration over time is paralleled by the reduction of TH+ dopaminergic neurons. It is well-known that the neurotoxin MPTP damages dopaminergic neurons in the SN already shortly after application, which has been demonstrated by a reduced amount of TH+ cells [23,24,31]. In our study, we confirm the MPTP-induced decrease of TH activity in dopaminergic neurons of the SN. Although the amount of TH+ neurons is already significantly reduced three days after MPTP treatment, MRE parameters are changed not until 6dpi. Importantly, TH activity has been shown to decrease first, followed and paralleled by a “real” reduction in the number of neurons after MPTP treatment [32]. We confirm this observation by the quantified number of DAPI+ cells, which correlates with the changes in viscosity. This means that the changed loss and–to a lesser extent—storage modulus in our study are representative for the dopaminergic neurodegeneration in the SN. Moreover, our findings are in accordance with the hypothesis that a decrease of viscoelastic properties of the adult brain is mainly based on the reduced number of neuronal cells, which has first been investigated in a murine stoke model as an example of disturbed brain structure [17].

In line with a decreased brain stiffness in APP-PS1 mice, modelling AD [18], our results support the assumption that viscoelastic properties in neurodegenerative diseases decrease in the mainly affected area and can be correlated with histopathological changes in our animal model for PD. As investigated here, the neurotoxin MPTP leads to different viscous and elastic changes in adult mice depending on the studied brain area. Therefore, we conclude that alterations in MRE parameters following MPTP treatment are highly region-specific.

The size of the processed ROI in the SN is a relevant factor in our study. While in previous animal studies, MRE changes have been observed in the whole brain [16], in one hemisphere [17], in the hippocampus [1] or the cerebellum [15], we processed our MRE data from a smaller brain region. However, our correlating histological findings imply that the MRE setup is eligible to detect viscous and elastic alterations even in small brain areas such as the SN.

Besides dopamine depletion, MPTP provokes an inflammatory response. It has been shown, that the neurotoxin initially leads to a higher amount of microglia and oligodendrocytes in the SN and hippocampus, which diminishes over time after treatment cessation [1,21,23,33]. This course of inflammatory reaction in response to MPTP is also seen in our study. However, the initially elevated amount of Iba+ cells in the SN is not reflected in MRE parameters. Similar observations have been made before in the hippocampus by Klein et al. [1]. Thus, the present data suggest that MRE may not be suitable for detecting elevated amounts of microglia and macrophages or oligodendrocytes as a sign of a transient local inflammation at least with regard to particular structures as the SN and hippocampus. Therefore, the present data further support the hypothesis that neuronal cells likely constitute the mechanical backbone of the adult brain. However, the biomechanical properties of tissue not only depend on mere cell numbers of one neural cell type but also on other cell types, the network neural cells build by cross-linking and branching and their interaction with the extracellular matrix. Even though neuronal cells have been identified to playing a key role in viscoelasticity, important influence from other cell types, networks and interactions cannot be excluded.

In the present study, female mice were used. Human studies have revealed that female brains are stiffer than brains of age-matched male counterparts [7,9]. This raises the question, if the observed changes predominantly in the viscous properties of SN tissue following MPTP-induced neurodegeneration are stable across sex in mice or if differences as in the mentioned human studies can be found. We have successfully established the MPTP mouse model in females to study the dopamine dependency of functional neurogenesis in the hippocampus and SN [23,24,34]. Thus, the present study can also be compared to our previous MRE study in MPTP-treated female mice [1].

In summary, we demonstrated the feasibility of MRE to sensitively detect viscoelastic changes in small and specific brain regions within an animal model for PD. Furthermore, we contribute to the investigation of the missing link between histopathological alterations and observed biomechanical constants in the SN, by demonstrating that changes in the amount of dopaminergic neurons in the SN of MPTP-lesioned mice are detectable by MRE. Thus, MRE is highly sensitive for the observation of local viscoelastic changes in particular brain regions adding to the understanding of how altered histopathological conditions influence biomechanical parameters of brain tissue that are changed under pathological conditions. This will help to establish MRE as a new potential instrument for clinical evaluation and diagnostics of neurodegenerative diseases.

## Supporting Information

S1 Table Results of MRE measurement in the substantia nigra, the midbrain and the hippocampus. (PDF)

S2 Table Results of histological cell count in the substantia nigra, the midbrain and the hippocampus. (PDF)
