# Supplementary material for: Dopaminergic Neurodegeneration in the Mouse Is Associated with Decrease of Viscoelasticity of Substantia Nigra Tissue
Source: PLoS One. 2016 Aug 15;11(8):e0161179. doi: 10.1371/journal.pone.0161179 (PMC4985068; doi:10.1371/journal.pone.0161179)
Supplement: S2 Table — (PDF) [file pone.0161179.s002.pdf]

S2 Table. Results of histological cell count in the substantia nigra, the midbrain and the hippocampus.

Post-injection day (dpi)	TH+ cells in the substantia nigra					Iba-1+ cells in the substantia nigra				
-3	1752	2202		1776	2340	16470	14280		29610	17700
3	1080	342	1026	576	1254	20280	18570	16350	16140	16260
6	972	1080	828	1080	432	15090	17400	15480	17790	15000
10	642	1188	1464	444	1092	15480	16710	13920	17070	14610
14	1146	1212	1146	546	924	15480	16710	13920	17070	14610
18	1044	1116	1260	1104	456	16320	13770	14120	14700	14370

	DAPI+ cells					
Post-injection day (dpi)	Substantia nigra		Midbrain		Hippocampus	
-3	217733	231666	1718400	1951200	1540800	1230000
3	228833	188833	2107200	1766400	1204800	1202400
6	155666	172500	1273200	1294400	1378800	1557600
10	182833	186333	1591200	1572000	1068000	996000
14	179500	218500	999600	1917600	1240800	946800
18	165500	187833	1611600	1530000	1262400	1122000

Dopaminergic neurodegeneration in the mouse is associated with decrease of viscoelasticity of substantia nigra tissue.
Hain EG, Klein C, Munder T, Braun J, Riek K, Mueller S, Sack I, Steiner B.
